# Teaching improvement science to paramedicine students: protocol for a systematic scoping review

**DOI:** 10.1186/s13643-018-0910-7

**Published:** 2018-12-20

**Authors:** Robin Pap, Louis Shabella, Alan J. Morrison, Paul M. Simpson, David M. Williams

**Affiliations:** 1 School of Science and Health, Western Sydney University, Locked Bag 1797, Penrith, NSW 2751 Australia; 2 Ambulance Service of New South Wales, Locked Bag 105, Rozelle, NSW 2039 Australia; 3 Institute for Healthcare Improvement, 53 State Street, 19th Floor, Boston, MA 02109 USA

**Keywords:** Paramedicine, Improvement science, Teaching methods

## Abstract

**Background:**

It is now more important than ever to equip paramedic students, the likely future managers and leaders of ambulance services, with the knowledge and skills of improvement science. Effective teaching requires a range of teaching methods that will engage students actively in learning. Although the array and effectiveness of methods used for teaching improvement science to clinicians and healthcare students has been systematically reviewed, the evidence regarding the specific sub-group of paramedicine students has yet to be fully explored and synthesized in the literature. The aim of this scoping review is to systematically explore and critically appraise the current state of evidence regarding strategies to teach improvement science to paramedicine students.

**Methods:**

A number of electronic databases (i.e., PubMed, CINAHL, Embase, Scopus, and ERIC) and gray literature (i.e., ProQuest Dissertations and Theses, Open Thesis, and Networked Digital Library of Theses and Dissertations) will be searched for published and unpublished evidence regarding teaching improvement science to paramedicine students. Included studies will undergo narrative synthesis to examine similarities and differences and to identify patterns, themes, and relationships (e.g., how and why certain teaching strategies or methods have worked in achieving desired learning outcomes (or not) and factors that might have influenced this).

**Discussion:**

To the knowledge of the authors, this is the first review that will systematically explore and critically appraise the current state of research evidence regarding strategies to teach improvement science specifically to paramedicine students. It is anticipated that the findings of this review will help to inform academics, developers of paramedicine teaching curricula, and researchers who are planning projects in this area.

**Systematic review registration:**

Scoping reviews are currently not eligible for registration on the international prospective register of systematic reviews (i.e., PROSPERO).

**Electronic supplementary material:**

The online version of this article (10.1186/s13643-018-0910-7) contains supplementary material, which is available to authorized users.

## Background

While a definition of paramedicine is still a subject of debate within this young healthcare discipline [1], it is clear that one of the core functions of paramedics is the provision of effective and safe out-of-hospital care. The relatively young age of the paramedicine profession and associated lack of research capacity, combined with the complexities of conducting data collection in a mobile health care setting, have meant that out-of-hospital practice has generally been based on relatively weak evidence [2–5]. However, research progress is being made and has real and future potential to substantially expand and raise the evidence for out-of-hospital systems and care [4, 6, 7]. Besides the production of evidence, one of the most important challenges for any healthcare service in this century is the implementation of research evidence into routine practice [8]. Ambulance services are no exception, and thus, with the evidence base expected to grow as well as pressure to function in progressively complex and demanding environments [9], the success of out-of-hospital care systems is becoming increasingly dependent on effective implementation and broader quality improvement strategies.

Successful improvement efforts rely not only on the knowledge, experience and intuitions of subject matter experts, but, to be most effective, these insights must be framed scientifically and tested [10]. Therefore, in modern healthcare services, the unceasing efforts to advance the quality of care and to find effective solutions to problems are underpinned by a science of its own domain—improvement science. It entails system thinking, understanding variation, psychology of change, and the theory of knowledge that are applied to improve the performance of processes and organizations [10]. Improvement science is an evolving field which can be considered the scientific foundation of quality improvement, exploring how it can be best conducted [11]. It uses robust scientific methods to understand and evaluate the quality improvement process. In principle, improvement science aims to ensure that quality improvement efforts are based as much on evidence as the best practices they seek to implement [12].

Quality improvement is not new to the health services though and has its roots in the work of Avedis Donabedian, a physician and researcher most famous for the development of the widely applied Donabedian Model [13]. Further evolution of quality improvement in health care was greatly influenced by other industries, most notably manufacturing [14]. W. Edwards Deming, significantly contributed to what became known as total quality management. In the 1990s, Toyota’s *Lean* and Motorola’s *Six Sigma* methods influenced how quality was managed in health care and contributed towards a shift from quality assurance to continuous quality improvement [14, 15]. In 1999, the Institute of Medicine published *To Err is Human: Building a Safer Health System: Building a Safer Health System,* a report on adverse events in health care [16]. Similar research was conducted in countries other than the USA with equally concerning occurrence rates [17, 18]. Research into adverse events in health care gave patient safety and quality improvement further momentum and placed increased pressure on health care organizations to demonstrate that patients could trust in the safety and quality of the services they provide.

Traditionally, ambulances services had been considered predominantly as a call-handling and transportation service, encompassing some aspects of pre-hospital care [19]. This operational focus combined with the paucity of evidence for clinical interventions has contributed to paramedicine somewhat lagging behind in applying improvement methods compared to the hospital-based healthcare services [20–22]. A scoping review of quality indicators for out-of-hospital care suggests that a specific definition of quality in the context of paramedicine remains elusive and many metrics used to evaluate it remain focused on traditional ideologies [23]. A recent study investigating the adoption of quality programs in ambulance services throughout the USA found a high degree of variability in the tracking of performance measures and demonstrated the need for better access to quality improvement tools, guidance on how to adopt best practice, and staff development [24]. Timely conveyance of patients with urgent and emergency care needs to an appropriate health care facility remains a primary function of modern ambulance services. However, the scope of out-of-hospital care and coverage that ambulance services provide has evolved significantly [25–29]. Although modern ambulance services can simply be seen as one part of the larger healthcare system, out-of-hospital and mobile health care remains unique in many ways [20], not least of which is the physical environment in which it is provided. These factors may make the application of general improvement principles challenging, highlighting the need for ambulance service leadership to have the knowledge and skills of scientific quality improvement. In other healthcare disciplines, it has been argued that improvement science is now as essential to good practice as the human life sciences that is taught during undergraduate training [30].

In light of the growing body of evidence for out-of-hospital systems and care, the need for this evidence to be implemented, as well as the required scientific approach to do this effectively, it would appear that it is now more important than ever to equip paramedic students, the likely future managers and leaders of ambulance services, with the knowledge and skills of improvement science. Effective teaching requires a repertoire of teaching methods that will engage students actively in learning [31]. Although the range and effectiveness of methods used for teaching improvement science to clinicians and healthcare students has been systematically reviewed in other healthcare disciplines such as medicine and nursing [32–35], the evidence regarding the specific sub-group of paramedicine students has yet to be fully explored and synthesized in the literature. The aim of this scoping review is to systematically explore and critically appraise the current state of evidence regarding strategies to teach improvement science to paramedicine students. To achieve this overall aim, the review will:

1. Systematically search both published and gray literature to identify evidence concerning methods for teaching the theories and practices of improvement science to paramedicine students;
2. Map key concepts (e.g., publication date, geographical location, teaching methods, reported outcomes) to gain insight into the extent, range, and nature of research activity in this area;
3. Provide a critical narrative of the current teaching methods, identifying successes and challenges; and
4. Identify questions for future research regarding strategies for teaching improvement science in paramedicine education.

## Methods

There are a number of reasons why a scoping review, rather than a more traditional type of systematic review, might be conducted [36]. In the current study, the scoping review methodology was considered most suitable because it aims to explore and examine the extent, range, and nature of research activity in the area of interest and, in doing so, identify research gaps in the existing literature. Scoping reviews may be especially relevant to disciplines or areas with incipient evidence as they include a variety of study designs from both published and gray literature [37]. Quality improvement science, especially applied in the paramedicine discipline, is an emerging concept and unpublished sources which do not meet rigorous academic standards may still provide important information and insights. Beyond underpinning future systematic review or research, scoping reviews can also inform practice [38]. This scoping review could potentially provide a considerable evidence base to aid in the development of teaching curricula and selection of teaching methods in paramedicine programs.

This review protocol was developed using the PRISMA-Protocols (PRISMA-P) 2015 checklist (Additional file 1: Table S1) [39], and the final review output will adhere to the Preferred Reporting Items for Systematic Reviews and Meta-Analysis (PRISMA) statement [40]. Considering the iterative nature of scoping review methodology, any amendments to the protocol will be reported explicitly (i.e., date, description, and rationale) in the final review output. Currently, scoping reviews are not eligible for registration on the international prospective register of systematic reviews (i.e., PROSPERO). This review was exempt from human research ethics review at Western Sydney University.

### Inclusion criteria

The aim of this scoping review is to systematically explore and critically appraise the current state of evidence regarding strategies to teach improvement science to paramedicine students. To meet this aim, any evidence that meets the following PICOCS (participants, interventions, comparators, outcomes, context, study designs) and study type criteria will be considered for inclusion.

#### Participants

Students studying towards a qualification in paramedicine and/or academic staff teaching paramedicine, both full-time and part-time students, will be included. Academic staff will be defined as teachers or scholars (e.g., tutors, lecturers, professors) in permanent or casual employment on a full-time or part-time basis.

#### Interventions

Any teaching strategy or learning activity used to facilitate the learning of concepts, strategies, methods, or techniques of improvement science. A teaching strategy will be defined as the structure, system, methods, techniques, procedures, or processes that academic staff use to deliver relevant information. These teaching strategies, such as lectures, seminars, printed materials, or audio-visual materials, are employed or provided by academic staff to assist student learning. Learning activities refer to the academically guided instructional tasks or assignments for students. These are student activities, such as group discussions, problem-solving workshops, or quality improvement projects.

#### Comparators

No comparator required.

#### Outcomes

Articles that report any outcome related to the effectiveness of teaching strategies (e.g., structured interviews with students, student satisfaction surveys, tests and exams, content analysis, peer observation feedback or specific outcome measures).

#### Context

One of the main aims of this review is to inform paramedicine curriculum developments and potential primary research projects in this field in Australia. The education and training of the paramedicine workforce in Australia is in the final stages of its transition from vocational training programs to university-level education programs [7]. However, considering that in many other countries paramedicine education is offered through non-university training courses, these will be included. For the purpose of this scoping review, undergraduate and postgraduate university programs as well as non-university programs will be considered.

#### Study designs

Any study design (i.e., quantitative, qualitative, or mixed methods) as well as opinion pieces, commentaries, letters and editorials. Industry reports, position papers, or program reports will be included. Review articles will be excluded, but relevant papers will be used to crosscheck for primary papers. Personal blogs and social media posts will be excluded.

### Search strategy

The search strategy will aim to find both published and unpublished studies. As recommended by the Joanna Briggs Institute (JBI) [38], a three-step search strategy will be utilized. An initial limited search of PubMed and CINAHL (Cumulative Index to Nursing and Allied Health Literature) will be undertaken followed by analysis of the text words contained in the title and abstract, and of the index terms used to describe articles. A second search using all identified keywords and index terms will then be undertaken across all included databases. Thirdly, the reference list of all identified reports and articles will be searched for additional studies. Only English language papers will be included for pragmatic reasons. Searches will not be limited by date. The databases to be searched include PubMed, CINAHL, Embase, Scopus, and ERIC (Educational Resources Information Centre). The search for unpublished studies will include ProQuest Dissertations and Theses, Open Thesis, and NDLTD (Networked Digital Library of Theses and Dissertations) as well as backtracking of references. Furthermore, experts in the field of study will be consulted to identify potential literature for inclusion. Experts will be contacted through the professional networks of the authors.

The search terms will likely be comprised of three facets [1]: terms to describe paramedicine, [2] terms to describe improvement science, and [3] terms to describe teaching strategies. The search terms will be developed by the principle investigator (RP) and one co-investigator (LS), with intellectual input from the rest of the review team (AM, PS, DW) and in consultation with a librarian. Where available, recommended search filters, such as one for paramedicine recently developed by Olaussen et al. [41], will be consulted. Details of the final search terms use will be included in the review output.

### Data management

All search results will be exported to Mendeley (Mendeley Ltd., 2018), an online reference management system. Mendeley will be used to delete duplicate records as well as to screen and share records between reviewers. Microsoft (MS) Office Excel (Microsoft Corporation, 2018) will be used for the data extraction process.

### Study selection

Study selection will be undertaken in accordance with the above inclusion/exclusion criteria. Initially, titles and abstracts will be screened against the eligibility criteria. If potentially eligible, the full text of the papers will be read to determine whether the article should be included in the review. If additional information is required to resolve queries about eligibility, the principle investigator (RP) will attempt to obtain this information via email from the respective corresponding author. Any non-responses will be reported anonymously in the final report. The principal investigator (RP) will undertake all selection stages, and 20% of all papers will be double-screened by a second reviewer (LS). Disagreements between the two reviewers will be discussed. Should reviewers not reach consensus, a third reviewer (AM) will be involved in the process to make a final decision on inclusion or exclusion. Reviewers will not be blinded to the journal title, study authors or associated institutions. A PRISMA flow diagram will be presented in the final output to detail search results and selection process.

### Data extraction

A standardized data extraction form will be developed a priori by RP and LS. This form will be published as an appendix in the final review output. Data from included articles will be extracted by the principle investigator (RP). To assess risk of error in data extraction, 20% of extracted data will be verified by a co-investigator (LS). Any disagreements between the reviewers will be discussed. Should reviewers not reach consensus, a third reviewer (AM) will be involved. If data is unclear, the principle investigator (RP) will attempt to obtain this clarification via email from the respective corresponding author. Any non-responses will be reported anonymously in the final report. Data to be extracted will likely include the following:

1. Study characteristics: author(s), publication year, title, place of publication, national setting, study aims/objective(s)/research question(s), study design, sample size, inclusion/exclusion criteria, recruitment method, data collection method, data analysis method and funding source/body.
2. Participant characteristics: student age, study load (part-time/full-time), and year of study.
3. Teaching strategy characteristics: curriculum (compulsory versus elective), teaching duration and spread (e.g., year, semester, module, hours per week) knowledge domain [42] (health care as process/system; variation and measurement; customer/beneficiary knowledge; leading, following, and making changes in health care; collaboration; social context and accountability; developing new locally useful knowledge; professional subject matter) and teaching method (e.g., lectures, group discussion, case study, project, web-based learning).
4. Outcome characteristics: student feedback (structured interviews, satisfaction surveys, etc.), before-after test/exam results, content analysis, and peer observation feedback.

During data extraction, it may become apparent that additional unforeseen data can be usefully charted [38]. Therefore, a pilot data extraction will be undertaken by RP and verified by LS. The charting process will be iterative in nature, enabling the principal investigator to update the data extraction form.

### Quality assessment

Congruent with the aim of a scoping review to provide an overview of the existing evidence regardless of quality [38], studies will not be excluded based on quality. However, to inform discussion around the overall “strength of the evidence” in this area, each paper will be scored for the strength of the findings in line with the system developed by the Best Evidence Medical Education (BEME) Collaboration [43].

### Data synthesis

The final review output will present extracted data from the included papers in diagrammatic and/or tabular form. Visual representation of appropriate form(s) will be provided in the results section to support the mapping of the extent, range, and nature of research activity in the area. Quantitative, qualitative, and mixed methods findings will be subjected to a narrative synthesis and the review output will present this in a descriptive format that aligns with the objectives and scope of the review. The narrative synthesis will seek to investigate similarities and differences between studies to explore patterns, themes, and relationships and propose explanations for findings, e.g., how and why certain teaching strategies or program have worked or have not worked. The synthesis will also assess the strength of evidence using the BEME grading system. Data synthesis will be undertaken by the principal investigator (RP) and discussed amongst the review team (LS, AM, PS, and DW) for validation.

## Results

Not applicable.

## Discussion

To the knowledge of the authors, this is the first review that will systematically explore and critically appraise the current state of research evidence regarding strategies to teach improvement science specifically to paramedicine students. It is anticipated that the findings of this review will help to inform academic staff, developers of paramedicine teaching curricula, and researchers planning projects in this area.

## Additional file

Additional file 1: **Table S1.** PRISMA-P (Preferred Reporting Items for Systematic review and Meta-Analysis Protocols) 2015 checklist: recommended items to address in a systematic review protocol*. (DOCX 33 kb)

## Abbreviations

BEME: Best Evidence Medical Education
CINAHL: Cumulative Index to Nursing and Allied Health Literature
MS: Microsoft
NDLTD: Networked Digital Library of Theses and Dissertations
PICOCS: Population, intervention, comparison, outcome, context, study design
PRISMA: Preferred Reporting Items for Systematic Reviews and Meta-Analysis
PRISMA-P: Preferred Reporting Items for Systematic Reviews and Meta-Analysis Protocols
PROSPERO: International Prospective Register for Systematic Reviews.
